# Experiences of new diagnoses among HIV-positive persons: implications for public health

**DOI:** 10.1186/s12889-022-12809-6

**Published:** 2022-03-18

**Authors:** Adobea Yaa Owusu

**Affiliations:** Institute of Statistical, Social and Economic Research (ISSER), University of Ghana, P. O. Box LG 74, Legon, Ghana

**Keywords:** Comorbidities, Ghana, HIV/AIDS, Depression, Psychosocial outcomes, Qualitative research, Experiences of new HIV-positive diagnoses, Hopelessness theory of depression

## Abstract

**Background:**

Ready acceptance of experiences of new diagnoses among HIV-positive persons is a known personal and public health safety-net. Its beneficial effects include prompt commencement and sustenance of HIV-positive treatment and care, better management of transmission risk, and disclosure of the HIV-positive status to significant others. Yet, no known study has explored this topic in Ghana; despite Ghana’s generalised HIV/AIDS infection rate. Existing studies have illuminated the effects of such reactions on affected significant others; not the infected.

**Methods:**

This paper studied qualitatively the experiences of new diagnoses among 26 persons living with HIV/AIDS. Sample selection was random, from two hospitals in a district in Ghana heavily affected by HIV/AIDS. The paper applied the Hopelessness Theory of Depression.

**Results:**

As expected, the vast majority of respondents experienced the new diagnoses of their HIV-positive infection with a myriad of negative psychosocial reactions, including thoughts of committing suicide. Yet, few of them received the news with resignation. For the vast majority of respondents, having comorbidities from AIDS prior to the diagnosis primarily shaped their initial reactions to their diagnosis. The respondents’ transitioning to self-acceptance of their HIV-positive status was mostly facilitated by receiving counselling from healthcare workers.

**Conclusions:**

Although the new HIV-positive diagnosis was immobilising to most respondents, the trauma faded, paving the way for beneficial public health actions. The results imply the critical need for continuous education on HIV/AIDS by public health advocates, using mass media, particularly, TV. Healthcare workers in VCTs should empathise with persons who experience new diagnoses of their HIV-positive status.

**Supplementary Information:**

The online version contains supplementary material available at 10.1186/s12889-022-12809-6.

## Background

News of a newly-diagnosed HIV-positive status has the tendency to lead to negative psychosocial outcomes for persons living with HIV/AIDS (PLWHAs) [1–3]. In this paper, the concept of newly-diagnosed refers to the first time a respondent was informed of his/her HIV-positive status. Implications of this operational definition are discussed later on in the paper. Humans depict cognitive, emotional, and motivational deficits on hearing bad news and experiencing what is deemed uncontrollable events [4]. This is very likely to apply to PLWHAs particularly, when confronted with the news of being HIV-positive for the first time. Particularly, PLWHAs are known to mostly experience neurocognitive disorders associated with the infection [5], and often experience common mental disorders [6, 7]. This is especially the case when PLWHAs fairly expect or self-perceive negative social reactions such as spousal abuse, dismissal from employment, stigma and discrimination, among others [8]. Peterson and Seligman ([4], p. 347) christened these series of responses on such occasions as “learned helplessness phenomenon.”

There are numerous news reports of persons who have committed suicide shortly after an HIV-positive diagnosis. This includes even the case of a female physician from the Eket local government area of Akwa Ibom, Nigeria [9]. Previous researchers note that after the initial diagnosis of a disease believed to be life-threatening, and particularly incurable, patients are known to experience an immediate descent into several distressing psychological and emotional states of mind [1, 2, 10]. These range from disbelief to denial to being utterly scared and shocked. Some even think that their life is not worth living anymore and imagine that indeed, the disease has already taken a final toll on them [2, 3]. This brings to the fore the need to study the reactions of PLWHAs right after their initial diagnosis. Research and programme interventions on adaptation to a new HIV diagnosis provide personal and public health safety-nets and are thus needed [2, 11]. Such research and interventions are helpful in educating newly-diagnosed PLWHAs on HIV [1, 10] and promoting their health [1, 8, 12].

Acceptance of the news of an HIV-positive diagnosis is critical for several reasons [2]. Engagement in and sustenance of HIV treatment and care [1, 2], viral load (VL), CD4 counts [2, 13], and transmission risk [2, 11, 14] are affected by the reaction to the news of diagnosis. Reactions also affect perceptions of stigma and disclosure activity [2, 11]. Conversely, difficulties with accepting diagnosed HIV-positive status has serious potential negative consequences for individuals and the general public [15, 16]. From the angle of healthcare practitioners, better handling of new diagnosis of an HIV-positive status and related disclosure could greatly buffer the psychosocial and mental health of the newly-diagnosed PLWHAs, and help facilitate healthcare seeking and retention. These include the management of less traumatised disclosure, reduction of self-stigmatisation, and better management of romantic and family relationships [2, 14]. Others are the reduction of viral transmission [14, 17], and improvement in general public health and well-being of populations [1, 14]. Knowing the experiences of new diagnoses among HIV-positive persons is, therefore, likely to facilitate successful linkage and retention of such persons in healthcare for HIV [1, 2]. This is critical and is also known to be a significant gap in the HIV care continuum in some parts of the world, even the U.S. with all its known medical advancement [11].

Based on previous related literature, this paper primarily examines qualitatively the experiences of new diagnoses of 26 Ghanaian PLWHAs to the first news of their HIV-positive status. Second, it attempts to untangle the situations surrounding these experiences to determine what might have influenced such reactions. Third, the paper delineates the processes that particularly helped the PLWHAs transition from negative psychological reactions to showing positive reactions and seeking HIV treatment. Fourth, it adds to the core literature on managing experiences of new diagnoses of an HIV-positive status, and illuminates their importance to public health. It is hoped that this paper will facilitate a better acceptance of an HIV-positive sense of self which is known to aid PLWHAs to accept, adjust, and more effectively cope with their diagnosis. This will aid them to better manage the complexities of living with the infection [2, 18]. This paper specifies the knowledge to Ghana and more importantly, Ghana’s most HIV/AIDS-affected district [19, 20]. A thorough search on the experiences of PLWHAs’ new diagnoses in Ghana yielded no known results despite the fact that Ghana has a generalised HIV/AIDS epidemic: more than 1% of the residents have the infection [21]. This paper aims to fill that gap.

### Immediate reactions to news of HIV positive status in Africa

The literature on the immediate reactions to the initial diagnosis of HIV-positive status is also sparse, and a majority of what research there is comes from South Africa. Such findings overwhelmingly corroborate each other: immediately after being diagnosed HIV-positive, PLWHAs studied depict deep negative emotions. Visser et al. [22] studied the phenomenon in South Africa, using a semi-structured interview of 293 pregnant women who were undergoing HIV test during antenatal care. On hearing the news, those who tested positive were shocked, and got frightened that they would be abandoned and discriminated against.

Fabianova [23] undertook a longitudinal study in Nairobi, Kenya, on the psychosocial aspects of being PLWHA. When the respondents who visited VCTs were first informed of their HIV-positive status, 89% of them felt sad due to their HIV-positive status, 60% had feelings of fear and anxiety, 30% felt angry, 25% felt distressed, and 15% cried. Additional psychosocial behaviours exhibited by the respondents included grief, guilt, hopelessness, helplessness, anger, disbelief, self-blame or blamed others, and aggression towards a counsellor. Their sadness was mostly in reference to close relatives who had died of AIDS. Their fears related mostly to the loss of their social position. Other fears surrounded loss of life, ambition, sexual relations, independence, physical performance, and financial stability [23]. While Fabianova [23] observed from the extant literature that suicide is a common reaction for persons who are first informed of their HIV/AIDS status, her study found that less than 1% of participants attempted suicide on hearing of their confirmed HIV-positive status. Most of Fabianova’s ([23], p. 201) respondents already considered themselves to be “walking corpses” and even visualised their funeral and grave.

Fabianova [23] enumerated several explanatory factors that unpacked her respondents’ reactions to their initial diagnoses of being HIV-positive. These included gender, level of preparedness of a client in the pre-testing session, and type of sexual relationship(s) they were in. Others included levels of general knowledge of HIV/AIDS, and HIV/AIDS-related stigma in their community. Fabianova ([23], p. 199) discovered that males responded to the initial HIV/AIDS diagnosis with anger, disbelief, and aggression. The females cried, got shocked, “swallowed big lumps of air, saliva subconsciously, shook both their hands in refusal and blame [sic] the others almost immediately.”

Other key explanatory factors for Fabianova’s ([23], p. 199) respondents’ immediate reactions included concerns about “lack of immediate elaborate support structures”, extent of level of awareness about HIV/AIDS, level of HIV/AIDS-related stigma, availability of antiretroviral therapy (ART), and support groups to enable them move on with their lives. Feelings of guilt for the infection were explained by whether the individual felt his/her lifestyle exposed him/her to it, and type of sexual involvement they were engaged in. They felt guilty that they would infect a spouse if they were married. If they were in an unstable/non-married relationship, they did not feel guilty, and shared the blame with the casual sexual partner. Sixty-two percent blamed their partners or the environment with the excuse that they stayed loyal to their partners. Fabianova’s ([23], p. 200) respondents who tested on their own volition did so based on their own or a partner’s “failure”, poor health or “accidental happening”, or work commitments.

The theoretical framework adopted for this paper, the Hopelessness Theory of Depression, is grounded on depression. Depression is currently one of the five leading causes of the disease burden internationally, except in sub-Saharan Africa (SSA) [24]. Researchers note that depressive disorders and other common mental health disorders (CMDs--depression, anxiety and somatization) were critically linked to the Millennium Development Goals, particularly gender equity, poverty, HIV/AIDS, and maternal and child health [24, 25]. Importantly, depression is the most diagnosed psychiatric disorder among PLWHAs. Depression also serves as a risk factor for the progression of HIV/AIDS. In African settings, a growing appreciation of an important link between CMDs and HIV/AIDS has been established [7, 23] as well. HIV has unleashed “a significant strain” on mental health in Africa ([24], p. 61; [26]). The huge burden of HIV/AIDS in SSA accounts for 16% of depression in the sub-region [24]. Alternatively, a neurobiological association exists between HIV and CMDs: “the HIV virus has quite specific detrimental effects on neuronal function” ([24], pp. 61 & 65). Evidence-based reports in African settings and other developing nations (studies conducted in Sao Paulo, Bangkok, Kinshasa, Nairobi, and Ethiopia), found both more symptoms and a higher prevalence of depression among symptomatic PLWHAs than among non-symptomatic or HIV-negative persons [24].

#### Theoretical framework: the hopelessness theory of depression

The Hopelessness Theory of Depression is applied to this paper. It is a diathesis-stress theory which posits that organisms express some form of cognitive and emotional deficits after experiencing a bad event. The theory argues that three depressogenic inferential styles serve as risk factors of depression [27, 28]. These are the tendency to attribute a bad event to a global or stable cause; the tendency to perceive bad events as having many disastrous consequences; and the propensity to view oneself as flawed or inefficient [28, 29]. Making negative inference upsurges the possibility of hopelessness while feeling hopeless makes depression inevitable. With this explanation, the theory assumes hopelessness as a critical underlying factor to depression. Adding to the causal explanation, Seligman [30] stated that the symptoms, cure and prevention of a bad event also model depression. In societies like Ghana, where HIV is associated with nonconformity to societal expectations and/or sexual promiscuity, PLWHAs may be more exposed to adverse emotional and cognitive symptoms after receiving an HIV-positive report.

The application of the thesis of the Hopelessness Theory of Depression to HIV-positive populations in SSA is not new. Govender and Schlebusch’s [31] study in Kwa-Zulu Natal, South Africa, applied Beck’s Hopelessness Scale and Beck’s Depression Inventory [32] to their assessment of the correlation between depression, hopelessness, and suicidal thoughts in PLWHAs. Schlebusch and Govender [33] used the same inventories to study PLWHAs in a University-affiliated hospital in South Africa. Primarily, they studied the prevalence of risk of suicidal ideation in PLWHAs immediately after their first diagnosis. Kylmä et al. [34] also studied the full gamut/dynamics of the concept of hope (hope, despair, hopelessness) among PLWHAs. Their study yielded information on how PLWHAs’ perceptions of hope could facilitate their clinical care. The Hopelessness Theory of Depression is applied to the discussion of the findings.

## Methods

### Study setting and cultural context of LMKM

The LMKM, the catchment area for the study, is situated in the Eastern Region of Ghana. The region, one of ten at the time of data collection, had 2,633,154 residents by Ghana’s last Population and Housing census of 2010, making it the third most-populated region. The Eastern Region is mostly semi-urban [35]. The LMKM, one of 26 administrative municipalities/districts in the Eastern Region by the time of data collection, covers 12.4% of the region, with total land mass of 304.4 km^2^. The 2010 Population and Housing Census recorded 89,246 residents of the Municipality comprising 46.5% males and 53.5% females. Christians were 92.8%; other religious groups include Muslims and traditionalists [36]. The indigenes are ethnic Dangmes and speak Krobo. They are a patrilineal descent group, which means they inherit property through their father’s lineage.

### Sampling and data collection

This study used a “descriptive, multiple case study approach” ([2], p. 2; [37]). This method generates interviewees’ in-depth descriptions of their situations, views and realities regarding issues. This provides deep insights of their actions and choices [38]. In this study, the pool of cases of the individual respondents is considered a “multiple case study approach” ([2, p. 2; 37]). Additionally, the findings from the study municipality forms a case study. As Kutnick et al. [2] note, case studies are exceptionally useful in eliciting contextual situations, when they are important to a particular study. Examples are cultural, social and structural impediments (such as stigma, and fear) to post-diagnosis HIV care [37].

This paper analysed data from 26 (13 each from two hospitals studied) out of 38 PLWHAs interviewed qualitatively through personal interviews from June to July 2015. The data used for this study formed part of a large data set from a project which primarily studied the linkages between housing conditions and the reported health status of PLWHAs (see [39–42]). The implications of recruiting individuals further away from their diagnosis for this paper are addressed in the section on limitations. The interviews were conducted with the aid of a pretested semi-structured question guide. Table 1 has the key questions asked. Some of these key questions in Table 1 were probing questions that emanated from the main/initial interview guide, because it is open ended, as is the norm with data collection tools for qualitative studies. The initial sample of 38 comprised both males and females who were selected using random sampling [2] as part of a research project which primarily studied the nexus between the health status of PLWHAs in the LMKM and their housing conditions.

**Table 1:** Key questions asked

**Key questions asked**	
What are some of the things you went through when you were first told you had HIV?	
Probe: What was your reaction when you were told?	
What did you do when you were told you had HIV?	
Probe: Were you (greatly) disturbed? How did you feel?	
What was your experience when you were diagnosed with the HIV virus? What has been your experience thus far (since you were diagnosed with HIV)?	
When you were informed about your illness, what were some of the things you went through? Probe: What did you suffer? How? Why?	
Did/do you have any specific experiences that made you happy or sad?	
Probe: Don’t you have any specific experiences that have brought you joy or sorrow?	
How did you manage it?	
Since when were you diagnosed with HIV? For how many years have you been diagnosed with this disease?	
Did the doctors tell you to go and do the test?	
When you came to the VCT [voluntarily] were you told it wasn’t true?	
What services [are provided that] have been helpful?	
Are you currently on ART?	
Probe: If yes, for how long have you been on ART?	

Source: Authors’ fieldwork, 2015

The project comprised both qualitative and quantitative data collection. The qualitative interview guide has been submitted as Additional file 1. First, the study district, LMKM, was conveniently selected based on its lead, by the time of developing the project proposal, in having persons with HIV/AIDS in Ghana, due to which the government had focused on strengthening healthcare institutions and personnel in the district for the fight against HIV/AIDS. Second, two out of three health facilities in the study district were also conveniently selected—a government and quasi-government hospitals. These had been specially equipped by the Government since 2002, to manage HIV/AIDS cases, thus conveniently leaving out the other government hospital [39–42]. Respondents had come to HIV/AIDS Voluntary Counseling and Testing (VCT) Centres in the two hospitals--St. Martins de Porres Hospital in Agormanya and Atua Government Hospital in Atua, near Agormanya, for care. Third, respondents were initially selected conveniently for having been medically diagnosed of being HIV-positive prior to the study, after which they had to verbally self-confirm their status to the PIs of the study.

Interviewing took place in these VCTs. The VCTs operated only on weekdays. For each day during the weekdays, based on prior reconnaissance survey, 40 respondents were targeted for both qualitative and quantitative interviews from the daily list of attendees in each hospital. One-third of these were selected randomly and interviewed. If there were less than 40 people in a day, half of them were randomly selected for interviews. For each day fewer than one-fourth of our initial sample were males, we increased the chances of having males in the sample, by including every fourth male from the initial list of persons who did not make it to the list from which final sampling was done [see [20, 40, 42]. This is justified based on a preponderance of female PLWHAs in the study district [20, 40, 42], Ghana, and also, sub-Sahara Africa generally [40, 42–44]. Respondents were given unique codes to prevent being re-interviewed during the course of the study. Five male and female graduate student interviewers were assigned to each study hospital, including one qualitative interviewer.

Qualitative or otherwise, a respondent got randomly selected for an interview when a prior interview had ended, beginning with a random start from the list of assigned codes, till the selected list was exhausted in a day. Similar sampling arrangements were made for each VCT used. The random selection of the respondents was necessary to spread their selection over the designated one month of study, for purposes of having a variety of cases/stories over the study period, for richer analysis. Out of the estimated average of about 40 PLWHAs who would visit each VCT per day, it was necessary to randomly select some and leave others out, to facilitate spreading respondent selection over time as said already. Additionally, it was expedient to randomly distribute the respondents between the qualitative study and survey data collection approaches for the larger project, to avoid possible biases in the responses from these two approaches.

Respondents were males and females, 18 years or older, and confirmed their HIV-positive status to the principal investigators (PIs). The interviewing time for a respondent in the qualitative study ranged between 35 and 50 min. The language used for the interview was left to the respondent’s choice between Krobo, the indigenous language in the LMKM, Twi, the indigenous language spoken the most in Ghana, and English, Ghana’s official language due to its colonial past. With prior permission from the respondents, the interviews were audio recorded.

This paper uses the qualitative data from the project. The original sample of 38 for the initial qualitative data was primarily informed by previous literature on the need to reach saturation in qualitative interviews [20, 42, 45, 46] which was achieved subjectively after interviewing about 10 respondents in each of the two study sites [20]. Although thematic saturation is usually achieved with 30 qualitative participants [20, 45, 46], 10 respondents gave us thematic saturation at each VCT, because they were quite homogenous [20]. Previous studies [20, 38, 42, 45, 47] have defined thematic saturation as recurrence or repetitiveness of responses from qualitative respondents. Despite reaching saturation with a combined 20 respondents from both VCT centres, we continued interviewing up to 38 of them to meet an initial objective of spreading respondent selection over a month [40, 42], with the hope of having some variety of responses due to the case study nature. The sample of 26 for this paper were selected from the 38 because the rest did not respond to the primary question on how they experienced the new diagnoses of their HIV-positive status (see [2]).

### Ethical clearance and data quality approaches

Ethical clearance was sought from the Ethical Committee for Humanities at the University of Ghana, Legon **(**ECH 017/14–15**)**, and the Ethical Review Board of the Memorial University at St. Johns, Newfoundland, Canada. Permissions were also sought from the Ethical Review Committee of the Ghana Health Service (GHS-ERC: 02/11/14) for using institutions under its jurisdiction for the study, as well as from the Eastern Regional Directorate of the Ghana Health Service. The District Health Management Directorate of the LMKM and the administrators of the study hospitals responded to the written permissions with verbal permissions to undertake the study. The respondents were informed of the objectives of the study, assured of confidentiality, and their informed consent was sought. They were also informed that their participation was voluntary and they would not be penalised if they opted to turn down the interview at any time during the process.

One person declined to be interviewed, citing time constraints. No identifiable markers were used for the respondents (see [20, 39, 40, 42]).

### Data analysis

The data were transcribed verbatim, and reviewed by the author and team of transcribers to ensure accuracy. The adequacy of translation from the local languages to English was ascertained by holding several meetings between the author and the transcribers to interrogate the correct translations forth and back till a mutual agreement was reached on the correct translations.

Based on the deductive approach, the data analysis was done focusing on the objectives of the study [40]. Qualitative thematic content analysis, which enables interrogating narratives from all cases studied for a combined result [20, 40, 42, 48], guided generating themes and subthemes for this paper. In this paper, the qualitative thematic content analysis used also qualifies to be called a discourse or conversation analysis [49, 50]. It used a qualitative thematic coding [50, 51] of the different interviews/conversations with the respondents and observed the three key features of qualitative content analysis described by Schreier [50]. These are reducing the data, systematically analysing them, and applying flexibility in the analysis. The systematic analysis meant carefully examining every single aspect of the transcribed data and systematically describing their meaning [50, 52], as well as comparing and relating different parts of the data to one another [50]. NVivo version 11 professional software [53] was used to identify themes and sub-themes relevant to the objectives of the study and other relevant information. Again, back and forth meetings were held between the author and the team of two coding assistants, who coded the data independently, to mutually agree on their meaning and context.

These repeated processes of reviewing the data enhance intercoder reliability subjectively and improve the credibility/validity and reliability of qualitative data [40, 42, 54].

**Fig. 1:**
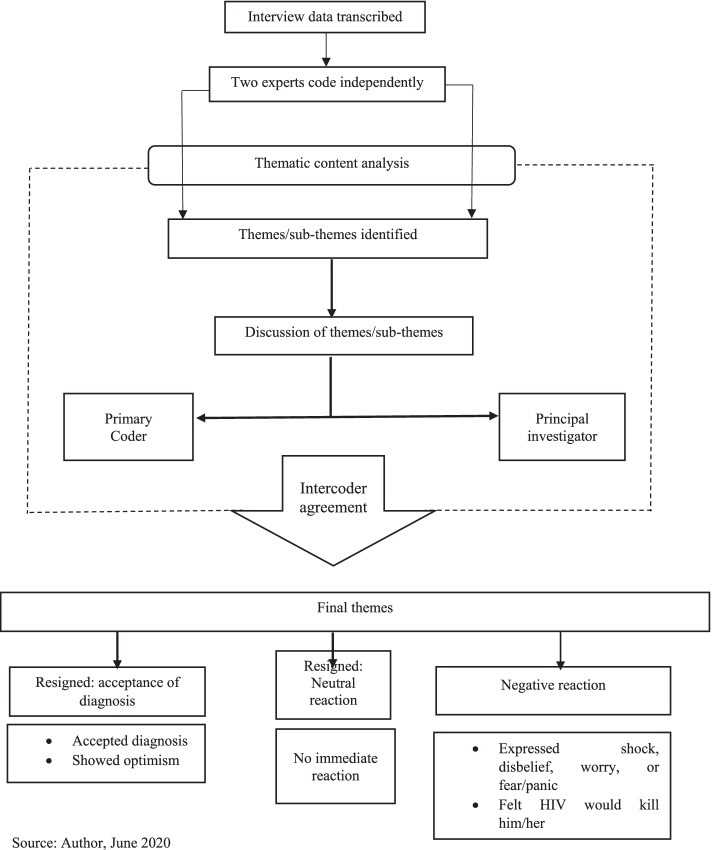
Data analysis workflow

Based on previous research [2, 11, 34] respondents’ experiences of the new diagnoses of their HIV-positive status were adjudged as negative if the respondent either expressed shock, disbelief, worry, fear/panic, felt the HIV infection would kill him/her, or immediately contemplated suicide. Respondents’ experiences of their new diagnoses were classified as resigned acceptance if they readily accepted the new diagnoses and/or showed some optimism instantly that they could survive the infection. Finally, their experiences of the new diagnoses were considered resigned neutral when the respondent indicated he/she showed no emotion upon receiving the first news of his/her HIV-positive status (Fig. 1 & Table 2).

**Table 2:** Experiences of new diagnoses among HIV-positive persons: major themes and subthemes

**Number**	**a) Experiences of new diagnoses**	**Classification of first reaction/final themes**
1	I became very worried and did not even know what to do. I even wanted to kill myself.	Traumatised, expressed negative reactions
2	When I heard it I was devastated and very worried.	
3	I was very frustrated and worried when I was told; in fact, I cried.	
4	It was very hard for me when I first heard it; in fact, I cried and cried.	
5	It worried me very much; in fact, I was very frustrated. I even decided to take poison so that I will die before my sickness becomes worse.	
6	In fact, I was heartbroken and I did not believe the nurses … The doctor … I didn’t believe him. He gave me a report to take to the hospital but I threw the paper away because I didn’t believe what he told me.	
7	I was very surprised and shocked when I was first told I had this disease. It was very heartbreaking.	
8	I was very worried at first.	
9	In fact, I was disturbed initially.	
10	I was disturbed but because they have drugs to treat it I was OK.	
11	I was disturbed, I was worried.	
12	I was really worried and disturbed.	
13	It has been a very bad experience … people may think when you have HIV it is promiscuity.	
14	I was disturbed, the first day I was told, I was greatly disturbed …. I was greatly disturbed and I said I will kill myself … I was always crying.	
15	I was really disturbed. That day I cried.	
16	When I was first diagnosed with HIV and was told I had HIV, I decided to break up with my wife because I was confused … It wasn’t easy … it wasn’t easy at all … I felt so bad, I felt very, very bad.	
17	Oh, I was worried but didn’t say anything.	
18	I was disturbed. I had to be disturbed because I didn’t expect it. I was disturbed.	
19	I was initially frightened that I would die because when you are told that you have this you would think that you were going to die.	
20	It seemed to be untrue.	
21	I was scared when I was told that I had the disease, so I did not want to be with anyone. When I was first informed about my status I would not even take medication.	
22	I can’t tell because I was ill for a long time so when I was told about it I only continued to take medications.	Resigned: expressed neutral reactions
23	I didn’t really do anything, as for sickness, its sickness so I didn’t really do anything.	
24	I accepted the result.	Resigned: accepted diagnosis
25	When I was told I was not perturbed because I knew people who were also taking the drug.	
26	There was nothing I could do about it.	
**b)**	**Influences on experiences of new HIV-positive diagnoses**	**Sway**
	**Major theme: Resigned—expressed neutral reactions**	Very few
	***Sub-themes***	
	• Had long illness prior to diagnosis	
	• Accepted illness as inevitable, and could happen anytime	
	**Major theme: Resigned—accepted diagnosis**	Few
	***Sub-theme***	
	I experienced persistent comorbidities; resultantly, I self-tested for HIV	
	**Major theme: Traumatised, expressed negative reactions**	Vast majority
	***Sub-themes***	
	i. *Most respondents:* I experienced comorbidities hence I voluntarily tested for HIV	
	ii. *Nearly half/slim minority:* spouse/partner was HIV+; resultantly: • I knew I had contracted the infection; • healthcare workers tested me for it	
	iii. *Few:* Spouse/partner had the infection so I tested voluntarily for it	
	iv. *Few:* I was pregnant; resultantly I got tested mandatorily at healthcare centre during antenatal care	
**c)**	**What facilitated transitioning to accepting HIV-positive status?**	**Sway**
	**Major theme: Resigned—accepted diagnosis or expressed neutral reactions**	Few
	***Sub-theme***	
	There were no need for transitioning for acceptance of diagnosis; readily embraced diagnosis	
	**Major theme: Resigned—Accepted diagnosis**	Few
	***Sub-theme***	
	We are not alone in this; we already know other PLWHAs	
	**Major theme: Resigned--expressed neutral reactions**	Very few
	***Sub-themes***	
	• Already had comorbidities; started treatment right away	
	• Sickness is inevitable; there was/is no need for me to worry about the HIV+ diagnosis	
	**Major theme: Traumatised, expressed negative reactions**	Vast majority
	***Sub-themes***	
	i. *Most important factor:* counselling/encouragement from healthcare workers to commence and adhere to treatment	Majority
	ii. *Second most important factor:* PLWHAs were aware of current medical breakthrough in treating HIV/AIDS which can prevent death from AIDS	Some/slim minority
	iii. There was no need to worry about the HIV+ diagnosis	Few
	• There is no need to contest/challenge medical diagnosis	
	• I had to face reality	
	• I lived by biblical principles and did not yield to suicidal ideation	
	iv. My mother-in-law emphatised with me and sent me to VCT	One person
	v. I have still not settled done to the HIV+ diagnosis	One person
	vi. Respondents gave no reason for their transitioning	Nearly a third of those who reacted negatively to the initial diagnosis

Source: Author’s fieldwork, June-July, 2015

#### Reflexivity

Reflexively, the author’s cultural and theoretical population health orientation admittedly had some influence on aspects of the research process, although these were minimal. These included the choice of the title. Being fully aware of the extent of stigmatisation culturally associated with HIV/AIDS in Ghana [20, 40, 42], the PIs of the project from which data for this paper emanates were curious about how respondents would experience the new diagnoses with the infection.

Nevertheless, given the author’s extensive training in the dogma and ethics of scientific research, particularly regarding the need for value-neutrality and objectivity [54], the author’s cultural and theoretical backgrounds did not otherwise influence the data analysis and interpretation. This was applicable to most of the research process as well.

## Results

### Socio-demographic characteristics of respondents

The respondents’ ages ranged from 25 to 68 years old. They were mostly females (80.77%, *n* = 21), primarily indigenous Dangmes (84.62%, *n* = 22), and generally had low socio-economic status. Also, they were mostly single (separated/widowed after cohabiting/never married/widowed), and they had been officially diagnosed HIV-positive for a range of six months to 12 years (Table 3).

**Table 3:** Socio-economic characteristics of respondents

Indicator	Details	Total (%)
Age	25–68 years Median age: 49 years Standard deviation: 10.94 years Mean age: 46.84 years	
Sex	Females: 21 (80.8%) Males: 5 (19.2%)	26 (100%)
Ethnicity	Indigenous Dangmes: 22 (84.6%) Others: 4 (15.4%)	26 (100%)
Employment status	Petty traders: 8 (30.8%) Farmers: 2 Seamstress/auto electrician: 3 Unemployed: 8 (30.8%) Labourer: 1 Apprentice: 1 Public sector workers: 2 Retiree: 1	26 (100%)
Level of formal education	None: 5 (19.2%) Less than basic school, i.e., less than current 9/former 10 years post nursery school: 10 (38.5%) Basic school: 7 (26.9%) Senior high: 1 Vocational: 1 Some College/College: 2	26 (100%)
Marital status	Single (separated/widowed after cohabiting/never married/widowed): 17 (65.4%) Married: 6 (23.1%) Cohabiting: 3	26 (100%)
Duration of HIV+ status	6 months-12 years Median: 5.0 years Mean: 5.43 years Standard deviation: 3.52 years One respondent: “less than a year ago”; s/he was not added to the calculation for the average years of HIV+ diagnosis	
Had biological children/young biological dependants at the time of diagnosis?	Yes: 18 (69.2%) No: 8 (30.8%)	26 (100%)
Had non-biological children/young non-biological dependants at the time of diagnosis?	Yes: 12 (46.2%) No: 14 (53.8%)	26 (100%)
HIV+ status of spouse/partner	Positive: 9 (34.6%) Negative: 4 (15.4%) Unknown/not tested: 13 (50.0%)	26 (100%)

Source: Author’s field work, June-July, 2015

### Thematic findings

#### Experiences of new diagnoses of HIV/AIDS

Table 2 summarises the experiences of new diagnoses among the respondents, upon hearing of their HIV seropositive status. There were negative and resigned reactions. Verbatim responses are identified with the type of hospital the respondent used: government or quasi-government.

##### Negative reactions

The data showed that the vast majority of the respondents seemed to have been very hard hit and traumatised by the news of HIV-positive test result, and expressed very negative sentiments. For these, the experiences of new diagnoses ranged from disbelief, to being very disturbed. Few mentioned the thought of killing themselves, incessant crying, and feeling that they would die from AIDS. Other reactions included being very frustrated, very surprised, very frightened, deep shock, deep worry, being heartbroken/devastated, self-blame, and avoiding going to hospital till conversion to AIDS (Table 2):

“*It was very hard for me when I first heard it; in fact, I cried and cried.”* **(**Government hospital).

*“In fact I was very frustrated. I even decided to take poison so that I will die before the sickness* [HIV] *becomes worse.”* (Government hospital).

*“I was very surprised and shocked when I was first told I had this disease. It was very heart-breaking.”* (Government hospital).

##### Resigned reactions

On the contrary, few respondents showed resignation towards the bad news. These comprised three who readily accepted the diagnosis and two who seemed neutral to it. From their responses, respondents who accepted their diagnosis seemed to have readily and consciously accepted/embraced the diagnosis of HIV-positive and encoded it rather smoothly as part of their identity.

*“When I was told I was not perturbed because I knew people who were also taking the drug”* [ARVs]. (Quasi-government hospital).

The very few respondents who seemed to have been neutral to their new diagnosis of HIV/AIDS seemed to have continued with their lives seamlessly on the spare of the moment they received the news, and did not take a break to react to the news of being HIV-positive.

*“I was ill for long time so when I was told about it I only continued to take medications.”* (Government hospital)**.**

Further analysis indicated that these experiences of the new diagnoses were influenced by certain background situations. These are discussed in the next section.

### Factors influencing experiences of the new diagnoses

For the very few respondents whose new diagnoses experiences were neutral, being ill for a long time before the HIV-positive diagnosis, and accepting that sickness is inevitable and could happen anytime, were what influenced their experiences of the new HIV-positive diagnoses:

“*… as for sickness, it’s sickness; you have less control over sickness, so I didn’t really do anything.”* (Quasi-government hospital).

Two of the few respondents who readily accepted their new diagnosed HIV-positive status said they experienced persistent comorbidities from the HIV infection. Resultantly, they voluntarily went to the VCTs to test for their HIV status:

*“I started falling sick and strange rashes kept appearing on my skin, so when I took it to the hospital, I was told it was the virus.”* (Government hospital).

*“I frequently fell sick … and it was not getting better so I came to the hospital myself … I came (willingly) to them to be tested when I realised I have changed. So they were even happy I walked to them myself to be tested.”* (Quasi-government hospital).

Including two multiple responses, the vast majority who responded negatively to the experiences of new diagnoses of HIV-positive status, mentioned four main situations that informed their experiences of the new diagnoses. Mostly, they suffered comorbidities from their infection.

*“For about seven months I was not feeling well. It even got to a point I lost consciousness. All forms of tests were ran on me … but they could not find exactly what was wrong...My boss advised me … so I went to the hospital for the test and the nurses said I was HIV-positive.”* (Government hospital).

Next, nearly half of the respondents who reacted negatively said a spouse/partner was HIV-positive and thus knew they had contracted the infection from them, and/or due to that, health personnel tested them also for the infection. Importantly, half of these PLWHAs did not know their partners’ HIV status prior to respondent’s diagnosis.

“*I was staying with my husband by then but he died so it was after he died that the doctors realised that it was this disease he died of … afterwards I was tested and diagnosed with this disease.”* (Government hospital).

Similar to those who readily embraced their diagnosis, a few of those whose new diagnoses experiences were negative were tested voluntarily based on their sexual partner’s infection. Likewise, several participants whose new diagnoses experiences were triggered by the onset of comorbidities said they voluntarily went for the testing. Additionally, for a slim minority of the latter, their conviction to go for the VCT was based on mass media (often television) announcements and discussions on the signs and symptoms of HIV/AIDS.

*“Already I suspected that my husband had all the symptoms that are discussed on TV and radio about this disease. He fell sick often and also coughed most of the time.”* (Government hospital).

*“Even though I wasn’t falling sick, I came to be tested willingly after they announced everybody should get tested …*.” (Government hospital).

*“… I used to fall sick frequently and that was when a lot of noise and adverts were made about this disease on television and radio...I went to the St. Martin’s hospital and told the doctors about my situation and he asked me go for an HIV test and that was when I got to know I had this disease.”* (Quasi-government hospital**).**

Finally, few (including two who gave multiple responses—primarily that a spouse/partner was HIV-positive) mentioned that they got tested when they were pregnant.

*“I was about to give birth to my second born … my husband is also HIV-positive.”* (Government hospital**).**

*“I was diagnosed when I was pregnant when my second born and I visited the hospital … Yes, [my husband is HIV-positive].”* (Government hospital).

### Factors that facilitated transitioning to accept HIV-positive self

For the few respondents who showed resignation towards the experiences of their new HIV-positive diagnoses, there was no need for transitioning to accepting an HIV-positive self. Two of these respondents who readily embraced the new HIV-positive diagnoses said they already knew persons who were HIV-positive and were taking antiretroviral medications (ARVs):

*“I realised I was not the only victim; many people are also victims …”* (Quasi-government hospital**).**

Two others said they already had comorbidities from AIDS and started treatment right after diagnoses:

“*I was ill for a long time so when I was told about it I only continued to take medications.”*

(Government hospital**).**

Another participant thought sickness is inevitable and thus did not need to worry about such diagnosis.

Nearly one-third of the respondents whose experiences of the new HIV-positive diagnoses were negative gave no response regarding what influenced their transitioning to accepting their new HIV-positive diagnoses. They mostly felt uncomfortable/reluctant talking about it. The rest mentioned what facilitated their transitioning more spontaneously and/or more readily after some amount of probing. The vast majority of the rest who experienced negative reactions said the most important help with their transitioning was counselling from health workers who encouraged them to initiate and continue treatment, with the assurance that if they did so, they would survive the infection.

*“… After I came here* [VCT] *and was advised and encouraged to see something to live for, I have been okay. They* [health personnel] *have been very friendly and encouraging. In fact, they have helped me a lot****.”*** (Government hospital).

“*They* [health personnel] *counsel us and tell us the fact that we have this virus does not mean our world has come to an end.”* (Government hospital).

*“… After being put on medication, going through counselling and tests, and being told what to do, I was hopeful that if you adhered to the medication you could live long.”* **(**Quasi-government hospital).

For one of these, her mother-in-law was the main person who empathised with her:

*“I was really worried and disturbed but the encouragement and advice from the nurses and my mother-in-law … has helped me. After I told her* [mother-in-law] *I had been diagnosed … she brought me here* [VCT] *to introduce me* [to a nurse]*, so she has been helpful.”* (Government hospital).

The second main issue that facilitated the transitioning for few of the respondents was their awareness of the current medical advancement in the treatment of HIV infection, due to which *“you would live long”* **(**Quasi-government hospital) despite the infection:

*“… because they have drugs to treat it, I was OK. Provided it will not cut short my life span I am happy.”* **(**Quasi-government hospital).

*“In the olden days when there were no drugs you thought you would die … after being told, so you become afraid, but now we know there are drugs available so if you are able to take your drugs you don’t have any problem.”* **(**Quasi-government hospital).

Three respondents alluded to accepting medical diagnoses, facing reality, and living by Biblical principles as facilitating their acceptance of their new diagnoses.

“*I accepted it because you can’t deny what a doctor says”* **(**Quasi-government hospital).

*“I was disturbed but I thought to myself that it had already happened* ***”*** **(**Quasi-government hospital)*.*

*“I didn’t kill myself because I am a Christian and the Bible speaks against that. I forgot about everything and decided to keep coming for the medications and now by the grace of God I have lived for over twelve years* ***.”*** **(**Government hospital).

There was, however, a lone-voice who said she had still not settled down to the reality of being HIV-positive after having been diagnosed three years prior and seeking treatment for one-year post diagnosis. Thus, she has not been consistent in getting treatment and is already feed up with seeking treatment:

*“It seemed to be untrue. From time to time I stopped taking the medication … I am fed up with coming to seek treatment. I have been treating the sickness for over a year but the symptoms recur after I see the doctor.”* **(**Quasi-government hospital).

This lone-voice mentioned having severe comorbidities which were probably due to starting healthcare for HIV two years after diagnosis. She also mentioned experiencing extreme discrimination and ostracisation both at home and in public, which no doubt, are linked to her comorbidities.

## Discussion

This section discusses the findings of the study, with a bearing on the tenets of the Hopelessness Theory of Depression. Fear has been associated with HIV since its discovery in the 1980s. Fear is mostly fueled by misconceptions associated with the virus which is mostly linked to death and stigmatisation [55]. Grounded on fear of death and a feeling of helplessness, a wide range of reactions are exhibited upon diagnosis or disclosure of an individual’s HIV-positive status. Although these reactions are both from the individual in question and significant others of this individual, the literature has mostly focused on the reactions of the persons affiliated with these individuals after disclosure [56, 57]. Indeed, very few studies explore the individual’s reactions upon diagnosis or testing positive for the virus [55, 58]. Based on this drawback and the known public health significance of such immediate reactions to HIV-positive diagnosis, this paper examined the experiences of new diagnoses of Ghanaian PLWHAs after hearing the “bad news.”

Importantly, a critical examination of differences between participants from each of the two hospitals studied shows that 11 respondents from the government hospital had negative reactions to the new experience of their HIV/AIDS diagnosis while ten in the quasi-government hospital did so. On the other hand, the experiences of two respondents from the quasi-government hospital was adjudged as “Resigned: accepted diagnosis” while one participant from the government hospital showed a ‘Resigned: accepted diagnosis.” There were no other differences in the participants’ reactions between the two study sites (Table 2). Rather, the difference between the respondents basically stemmed from the influences on their experiences of the new HIV-positive diagnoses discussed earlier (Table 2), which did not have a relationship with the particular hospital they received healthcare from. While previous researchers have articulated the lack of major differences in the findings from the respondents used for the qualitative study in the project [39, 41], they have further clarified that the respondents’ similar socio-bio-demographic background is mostly responsible for the lack of major differences in the findings [20, 39, 41].

The findings from this study have corroborated those of previous authors that receiving the news of newly-diagnosed HIV-positive status is often met with reactions that are “complex and multi-faceted” ([2], p. 12). Additionally, the findings that respondents had varied responses and a myriad of experiences to the new diagnoses of their HIV-positive status is in line with reports from several studies (example: [2, 58]). Most of the initial reactions of respondents in this study were very traumatising and discouraging. These included a few who contemplated suicide upon hearing the news; supporting Fabianova’s [23] findings in Nairobi.

Similar to previous research [55, 58], findings from this study indicate that though negative emotional and psychological reactions may occur upon learning of an HIV-positive status, respondents may also resign themselves to their fate—to accept or numb their feelings about the new disclosure of their HIV+ diagnoses. Likewise, this study found that respondents who were already exposed to HIV/AIDS needed no transitioning to self-acceptance of their HIV-positive status. This corroborates previous findings [55]. Nearly all the respondents settled down later, after experiencing the new diagnoses, initiated and continued with their healthcare for HIV/AIDS. This affirms previous assertions that such initial feelings regarding information on a newly-diagnosed HIV-positive status mostly fade away eventually [3, 23].

Again, the paper corroborates previous findings and highlights the buffering role of healthcare providers in moderating the experiences of new diagnoses of HIV-positive persons [1, 11]: the vast majority of the respondents who adapted to their HIV-positive status attributed it to counselling and support from healthcare personnel. This role of the healthcare workers in aiding respondents’ transition to accepting their HIV-positive selves affirms the documented importance of social support in ameliorating the otherwise negative effects of experiencing a health trauma [23, 59]. Research has identified HIV counsellors’ or health providers’ choice of words and emotions as crucial in determining peoples’ reaction to the initial diagnosis of HIV infection [55, 58]. When these emotions were hopeful and assuaging, patients were more likely to be calm and comforted, and vice versa [58].

The findings of this study underscore the fact that PLWHAs who experience new HIV-positive diagnosis will need to receive such interventions early [12]. These interventions are needed to give them hope in life to abate extreme psychosocial trauma that can be associated with experiences of new HIV-positive status [11, 60]. Per the findings of this paper, such interventions should include educating PLWHAs experiencing new HIV-positive diagnosis that new medical advances for HIV-positive infection make it possible for PLWHAs experiencing new HIV-positive diagnosis to lead normal lives and live long, if they seek early treatment and adhere to prescribed healthcare.

A fair number of the respondents mentioned being infected by their spouses/partners who were alive or deceased (also see [40]), implying that they probably were not using condoms and other modes to prevent HIV transmission from their sexual partners. This finding is not unlikely considering that Owusu [42] found that the PLWHAs studied were hardly using any form of protection against HIV with their sexual partners, whether in stable or unstable relationships. Two respondents were exceptions--they used condoms, but inconsistently [40]. Furthermore, Owusu [40] found that the unmarried or non-co-habiting PLWHAs had not disclosed their HIV-positive status to their sexual partners, with the exception of one respondent. PLWHAs who fail to disclose their status to their partners may have been living in a state of denial and or may fear/have feared the ramifications of disclosing their status. They may thus refuse to disclose as a way of attenuating the anticipated effect of disclosing, as previous authors have attested to [12, 15, 23].

Additionally, findings from this paper illuminate the importance of voluntary counseling and testing. Majority of the respondents mentioned having had comorbidities before they tested for their HIV-positive status. Other researchers have indicated that anticipated reaction influences decisions on voluntary testing for HIV [55, 58]. Previous research clearly notes potential barriers to voluntary testing for HIV in Ghana. These include the fear of stigma, discrimination and abuse, and possible dissolution of romantic relationships associated with being a PLWHA. Importantly, there is fear of the perception that an HIV-positive diagnosis is a death warrant [16, 61]. Antenatal-linked VCT is a policy strategy in Ghana for HIV control through a nationwide integration of VCT and antenatal care [62]. However, clients may disagree to it.

The findings from this paper mostly tally with the Hopelessness Theory of Depression’s core proposition; the respondents overwhelmingly perceived the experiences of their new HIV-positive status as translating into unwelcome consequences and negative inferences, as did respondents in other SSA settings [12, 23]. This plausibly led to depression and feelings of hopelessness among them. Also, this perspective may have underlain the self-blame by a few of them for being HIV-positive [28, 29]. Furthermore, this paper confirms the Hopelessness Theory of Depression’s propositions that underlying perceived bad situations and experiences which are attributed to internal factors lead to depression. Conversely, those ascribed to presumed external factors/influences give comfort/are reassuring [4]. In this study, the experiences of new HIV-positive diagnoses were mostly negative for respondents with internal factors such as having comorbidities and having a spouse/partner with HIV/AIDS.

In consonance with Peterson’s and Seligman’s [4] Hopelessness Theory of Depression’s propositions, respondents of this study said that external factors motivated them to take commendable actions such as going for VCT. These included the influence of health education through mass media, counselling by healthcare practitioners, and information regarding modern medical advancements which can help PLWHAs live without comorbidities, and possibly survive the infection. Furthermore, these factors facilitated their adaptation to their HIV-positive selves.

Nevertheless, this paper does not substantiate Peterson and Seligman’s [4] Hopelessness Theory of Depression fully. Contrary to their proposition, in this study, fewer respondents mentioned that a series of external factors such as knowing someone who has HIV/AIDS, and being given a near-mandatory HIV test at an antenatal clinic were what influenced their negative reactions. Conversely, other internal factors such as having comorbidities and having an HIV-positive romantic partner influenced few of the respondents to positively adapt to their HIV-positive diagnoses. In this study, therefore, the clear diathesis of external factors giving psychosocial comfort and internal factors unleashing mental discomfort in response to hearing the often unwelcome first news of being HIV-positive was not fully supported. This was also true of Assen et al.’s [12] study in Ethiopia.

Hence, this paper corroborates Govender and Schlebusch’s [31] synthesis of the Hopelessness Theory of Depression. These authors emphasise that the numerous internal and external challenges that face PLWHAs such as discrimination, stigmatisation, abuse, financial, marital, and healthcare challenges, among others, may [also combine to] have connotations for the loss of control over one’s life, fear of the future, and feeling of helplessness. As well, they may underlie the negative inferences which exacerbate the feeling of hopelessness and increase the likelihood of depressive symptoms stemming from experiencing new diagnosis of HIV/AIDS.

This paper adds to the knowledge on the personal and public health effects of experiencing new HIV-positive diagnosis. Particularly, it highlights the commendable public health effects of receiving new diagnosis for HIV-positive status and illuminates the role of social support in seeking and continuing healthcare for the diagnosis. Also, it attests to the role of healthcare workers and behaviour change communication, using mass media, in fighting the menace of HIV/AIDS. Furthermore, as articulated above, this paper did not fully support the conclusions of Peterson’s and Seligman’s [4] Hopelessness Theory of Depression’s framework. Rather, the paper contributes additional information to it in the form of an anti-thesis. Additionally, unlike Fabianova’s [23] study in Nairobi, this study did not find that the respondents acted with aggression towards counsellors who first broke the news of their HIV-positive status to them.

More importantly, this paper has navigated new frontiers in the body of knowledge in its thematic area of study. First, contrary to Fabianova’s [23] findings that some of her respondents attempted suicide, non-of the respondents in this study mentioned having attempted to take their lives, although a few of them revealed they had suicidal ideation. This may be due to the fewer respondents this study engaged as well as its cross-sectional design, compared to Fabianova’s [23] respondents, and the longitudinal approach to her study. Second, this study has newly articulated outstanding information on factors which facilitate PLWHAs’ transitioning to accepting and settling down to their experiences of new HIV-positive status. Third, unlike previous literature, this study has uniquely found that having comorbidities from HIV/AIDS was the primary reason that influenced the respondents to voluntarily test for their HIV-positive status. The uniqueness of this finding may be linked to the awareness that VCT of HIV/AIDS status is very rare in Ghana [63, 64].

### Limitations

The qualitative nature of this study better facilitates unearthing the complexities associated with experiences of new diagnoses among HIV-positive persons and learning about the adaptation process [2]. Also, the repeated data analyses this paper employed strengthens its reliability and validity. However, when interpreting these findings, some limitations should be considered. With HIV-positive status being a very sensitive issue, and highly stigmatised in Ghana [16, 65, 66], social desirability of responses may have influenced the findings [2]. Being a retrospective study, recall bias may also affect the reliability of the responses [2, 20]. This is with particular reference to the time lapse between the moment a respondent was newly-diagnosed with HIV/AIDS, as operationalised above, and the time of the study. Recruiting individuals further away from their diagnosis is a limitation; it could lead to recall bias. Furthermore, the study is cross-sectional and does not permit inference of causality [67]. Additionally, being a qualitative study with a fairly small sample size, the findings are not generalisable to non-respondents in the LMKM and also, Ghana as a whole [67].

Omona ([68], p. 181) posits that although random sampling is rare in qualitative studies, they are not out of place as they facilitate sampling the “desired number of individuals …” from a generated sampling frame, which he asserts improves the credibility of the sample (also see [69], p. 28). Yet, as hinted previously in this paper, random sampling in qualitative studies could pose some limitations. A careful search of the literature does not specify such limitations. However, the author guesstimates that random sampling in qualitative studies may rob the data of the quality of focusing on some key persons/texts of interest to the study which convenience sampling will provide. Lastly, based on the primary focus of the project, this paper did not undertake a diagnostic assessment for depression among the respondents. The paper is thus unable to ascertain if depression contributed to experiences of the new diagnoses of HIV-positive status among the PLWHAs studied.

## Conclusions

Evidence elsewhere suggests that upon receiving the initial news of an HIV-positive diagnosis, most people have strong psychosocial reactions [23, 55, 70]. Such reactions are also known to have very critical public health implication [1, 2, 14]. Yet little research has focused on the initial reactions of newly-diagnosed PLWHAs in SSA, particularly Ghana. Neither has the implications of their reactions for personal and public health been extensively studied [2, 11]]. This makes this study of the experiences of new HIV-positive diagnoses very timely. The personal and public health implications of one’s experience of new diagnosis of HIV-positive status is critically important for Ghana, which has a generalised HIV-positive infection [21].

Consistent with the literature, the vast majority of the respondents became extremely traumatised and immobilised when they experienced new diagnoses of their HIV-positive status. A few, however, more readily resigned to their HIV-positive identity. Regardless of their experiences of new diagnoses of HIV/AIDS, having comorbidities prior to diagnosis influenced their experiences the most. This was followed by having/having had a spouse/partner especially, and/or knowing someone who was HIV-positive, prior to their diagnoses. Next, respondents mentioned being influenced by health education through mass media, TV particularly, on signs and symptoms of HIV/AIDS. Importantly, health education, counselling, reassurance and empathy from healthcare workers provided hope. Furthermore, these facilitated their transitioning to settling down to self-acceptance of an HIV-positive status and continuing with healthcare. Finally, this paper concludes that to a large extent, the findings are applicable to the tenets of the Hopelessness Theory of Depression used as a framework for this study. Consequently, this paper found hopelessness as an important driving force to negative reactions towards one’s experience of his/her new HIV-positive diagnosis.

Conclusions from this paper have several public health significances. It highlights the continuous need for and strengthening of behaviour change communication on HIV/AIDS by the Ghana AIDS Commission and Ghana Health Service. This should emphasise its signs and symptoms, the need to seek early treatment, and adherence to prescribed ARVs. Strengthening the use of mass media, small groups, schools, churches/mosques and person-to-person channels in such endeavours is important. HIV/AIDS-related health promotion and education should also continue to emphasise prevention, but importantly, state that once infected, HIV/AIDS can be controlled; the infected person can live without comorbidities and need not succumb to the infection. Stakeholders should work harder towards educating residents in the LMKM, and for that matter, Ghanaians generally, about voluntary testing of HIV status. Most of the respondents in this study mentioned having had comorbidities from HIV/AIDS prior to their new diagnoses. Onset of comorbidities for HIV/AIDS prior to diagnosis and treatment can make the treatment expensive; it can also diminish the chances of surviving the infection severely [71, 72].

Given the high rates of HIV/AIDS in the study district and Region, the health education should also emphasise the need for persons whose sexual partners are HIV-positive—particularly those who show signs and symptoms of the infection, and all who engage in at-risk sex, to practice safer sex. Finally, the paper recommends increased social support and empathy for PLWHAs in LMKM particularly and in Ghana generally, from family, friends, neighbors, community leaders, healthcare professionals, and organised groups such as members of their religious affiliation, if any [20, 40, 42]. This study has unearthed social support as a critical moderating element in the transitioning of PLWHAs to integrating their HIV-positive self-concept, initiating, and adhering to prescribed healthcare. Families, social groups, and healthcare professionals should empathise with PLWHAs.

## Supplementary Information

**Additional file 1.** Qualitative/Indepth Interview Guide. Housing and Health Needs of Persons Living with HIV/AIDS Project. Open-ended qualitative indepth interview guide with sections A to G and ending with demographic/background data.

## Data Availability

The datasets used and/or analysed during this study are available from the author on reasonable request. The key questions for the study have been provided in the paper, in Table 1. The complete question guide used to collect the data has also been provided online as Additional file 1.

## Abbreviations

AIDS: Acquired immune deficiency syndrome
ART: Anti-retroviral therapy
ARVs: Antiretroviral medications
CMDs: Common mental health disorders
ECH: Ethics Committee on Humanities
ERC: Ethical Review Committee
GAC: Ghana AIDS Commission
GHS: Ghana Health Service
GSS: Ghana Statistical Service
HIV: Human immunodeficiency virus
LMKM: Lower Manya Krobo Municipality
PI: Principal Investigator
PLWHAs: Persons living with HIV/AIDS
RAs: Research Assistants
SSA: Sub-Saharan Africa
TV: Television
UCSF: University of California San Francisco
VCT: Voluntary counselling and testing
VL: Viral load

## References

[1] Chu C, Selwyn PA (2010). Diagnosis and initial management of acute HIV infection. Am Fam Physician.

[2] Kutnick AH, Gwadz MV, Cleland CM, Leonard NR, Freeman R, Ritchie AS, McCright-Gill T, Ha K, Martinez BY, Banfield A, Belkin M (2017). It’s a process: reactions to HIV diagnosis and engagement in HIV care among high-risk heterosexuals. Front Public Health.

[3] University of California San Franscisco (2018). Coping with HIV/AIDS: Mental Health. HIV in Site Project: the UCSF Center for HIV Information.

[4] Peterson C, Seligman ME (1984). Causal explanations as a risk factor for depression: theory and evidence. Psychol Rev.

[5] Alford K, Banerjee S, Nixon E, O’Brien C, Pounds O, Butler A, Elphick C, Henshaw P, Anderson S, Vera JH (2019). Assessment and management of HIV-associated cognitive impairment: experience from a multidisciplinary memory service for people living with HIV. Brain Sci.

[6] Stein DJ, Seedat S, Emsley RA, Olley BO (2005). HIV/AIDS in Africa—a role for the mental health practitioner?. S Afr J Psychiatry.

[7] Rivera-Rivera Y, García Y, Toro V, Cappas N, López P, Yamamura Y, et al. Depression correlates with increased plasma levels of inflammatory cytokines and a dysregulated oxidant/antioxidant balance in HIV-1-infected subjects undergoing antiretroviral therapy. J Clin Cell Immunol. 2014;5(6). 10.4172/2155-9899.1000276. 10.4172/2155-9899.1000276 PMC4321812 25674354

[8] Wanyenze RK, Kamya MR, Fatch R, Mayanja-Kizza H, Baveewo S, Sawires S, et al. Missed opportunities for HIV testing and late-stage diagnosis among HIV-infected patients in Uganda. PLoS One. 2011;6(7). 10.1371/journal.pone.0021794. 10.1371/journal.pone.0021794 PMC3130049 21750732

[9] Forum N (2011). A female doctor commits suicide after testing positive to HIV.

[10] Hosek SG, Lemos D, Harper GW, Telander K (2011). Evaluating the acceptability and feasibility of project ACCEPT: An intervention for youth newly diagnosed with HIV. AIDS Educ Prev.

[11] Moitra E, Chan PA, Stein MD (2015). Open trial of an acceptance-based behavior therapy intervention to engage newly diagnosed HIV patients in care: rationale and evidence of feasibility and acceptability. Behav Modif.

[12] Assen A, Molla F, Wondimu A, Abrha S, Melkam W, Tadesse E, Yilma Z, Eticha T, Abrha H, Workneh BD (2016). Late presentation for diagnosis of HIV infection among HIV positive patients in South Tigray zone, Ethiopia. BMC Public Health.

[13] Dodd PJ, Garnett GP, Hallett TB (2010). Examining the promise of HIV elimination by ‘test and treat’ in hyper-endemic settings. AIDS (London, England).

[14] Pettifor A, MacPhail C, Corneli A, Sibeko J, Kamanga G, Rosenberg N, Miller WC, Hoffman I, Rees H, Cohen MS (2011). NIAID center for HIV/AIDS vaccine immunology. Continued high risk sexual behavior following diagnosis with acute HIV infection in South Africa and Malawi: implications for prevention. AIDS Behav.

[15] Skinner D, Mfecane S (2004). Stigma, discrimination and the implications for people living with HIV/AIDS in South Africa. SAHARA J.

[16] Koka E, Ahorlu CK, Agyeman DK (2013). Social death through HIV and AIDS stigmatization and discrimination in Ghana: a case study of the central regional hospital, Cape Coast, Ghana. Advanc Appl Sociol.

[17] Miller WC, Rosenberg NE, Rutstein SE, Powers KA (2010). The role of acute and early HIV infection in the sexual transmission of HIV. Curr Opin HIV AIDS.

[18] Gwadz M, de Guzman R, Freeman R, Kutnick A, Silverman E, Leonard NR, Ritchie AS, Muñoz-Plaza C, Salomon N, Wolfe H, Hilliard C, Cleland CM, Honig S (2016). Exploring how substance use impedes engagement along the HIV care continuum: a qualitative study. Front Public Health.

[19] Lund R, Agyei-Mensah S (2008). Queens as mothers: the role of the traditional safety net of care and support for HIV/AIDS orphans and vulnerable children in Ghana. Geo J.

[20] Owusu AY, Laar A (2018). Managing HIV-positive sero-status in Ghana’s most HIV concentrated district: self-perceived explanations and theoretical discourse. Afr J AIDS Res.

[21] GAC (2017). Summary of the 2016 sentinel survey report.

[22] Visser MJ, Makin JD, Vandormael A, Sikkema KJ, Forsyth BW (2009). HIV/AIDS stigma in a south African community. AIDS Care.

[23] Fabianova L. Psychosocial aspects of people living with HIV/AIDS. In Social and psychological aspects of HIV/AIDS and their ramifications 2011. IntechOpen. doi: 10.5772/21148. https://www.intechopen.com/books/social-and-psychological-aspects-of-hiv-aids-and-their-ramifications/psychosocial-aspects-of-people-living-with-hiv-aids. Accessed 14 Oct 2019.

[24] Patel V, Stein DJ, Akyeampong E, Hill A, Kleinman A (2015). Common mental disorders in sub-Saharan Africa: the triad of depression, anxiety and somatization. The culture of mental illness and psychiatric practice in Africa.

[25] Miranda JJ, Patel V (2005). Achieving the Millennium Development Goals: does mental health play a role?. PLoS Med.

[26] Collins PY, Holman AR, Freeman MC, Patel V (2006). What is the relevance of mental health to HIV/AIDS care and treatment programs in developing countries? A systematic review. AIDS (London, England).

[27] Abela JR (2001). The hopelessness theory of depression: a test of the diathesis–stress and causal mediation components in third and seventh grade children. J Abnorm Child Psychol.

[28] Abela JR, Payne AV (2003). A test of the integration of the hopelessness and self-esteem theories of depression in schoolchildren. Cogn Ther Res.

[29] Waszczuk MA, Coulson AE, Gregory AM, Eley TC (2016). A longitudinal twin and sibling study of the hopelessness theory of depression in adolescence and young adulthood. Psychol Med.

[30] Seligman ME (1975). Helplessness. On depression, development and death.

[31] Govender RD, Schlebusch L (2012). Hopelessness, depression and suicidal ideation in HIV-positive persons. S Afr J Psychiatry.

[32] Beck AT, Weissman A, Lester D, Trexler L (1974). The measurement of pessimism: the hopelessness scale. J Consult Clin Psychol.

[33] Schlebusch L, Govender RD. Elevated risk of suicidal ideation in HIV-positive persons. Depress Res Treat. 2015;2015. 10.1155/2015/609172. 10.1155/2015/609172 PMC4603315 26491561

[34] Kylmä J, Vehviläinen-Julkunen K, Lähdevirta J (2001). Hope, despair and hopelessness in living with HIV/AIDS: a grounded theory study. J Adv Nurs.

[35] GSS (2012). Population and Housing census 2010. Final results.

[36] GSS (2014). Population and Housing Report: District Analytical Report 2010, Lower Manya Krobo Municipal.

[37] Yin RK (2013). Case study research: design and methods.

[38] Baxter P, Jack S (2008). Qualitative case study methodology: study design and implementation for novice researchers. Qual Rep.

[39] Tenkorang EY, Owusu AY, Laar AK (2017). Housing and health outcomes of persons living with HIV/AIDS (PLWHAs) in the lower Manya Krobo district, Ghana. J Health Care Poor Underserved.

[40] Owusu AY (2020). A gendered analysis of living with HIV/aids in the eastern region of Ghana. BMC Public Health.

[41] Tenkorang EY, Owusu AY, Laar AK, Yeboah EH (2019). Housing, psychosocial and adherence counseling among HIV+ persons in Ghana. Health Promot Int.

[42] Owusu AY (2019). Social contexts of living with HIV/AIDS in the eastern region of Ghana. Istanbul Univ J Sociol.

[43] NACP (2018). HIV sentinel survey report.

[44] Owusu, A. Y. Gender, Adolescents, and Achieving Sustainable Development Goals in Ghana. In A. Williams & I. Luginaah (Eds.). Gender Matters Globally: Geography, Health and Sustainability (Chapter 4). Routledge. Forthcoming. ISBNS: Hardback: 9780367743901; Ebook: 9780367743918; Future paperback: 9780367743925.

[45] Guest G, Bunce A, Johnson L (2006). How many interviews are enough? An experiment with data saturation and variability. Field Methods.

[46] Mason M (2010). Sample size and saturation in PhD studies using qualitative interviews. Forum qualitative Sozialforschung/Forum: qualitative social research.

[47] Sedziafa AP, Tenkorang EY, Owusu AY (2016). “… he always slaps me on my ears”: the health consequences of intimate partner violence among a group of patrilineal women in Ghana. Culture Health Sexual.

[48] Lieblich A, Tuval-Mashiach R, Zilber T (1998). Narrative research: reading, analysis, and interpretation.

[49] Krippendorff K (2004). Content analysis: an introduction to its methodology.

[50] Schreier M (2012). Qualitative content analysis in practice.

[51] Boyatzis RE (1998). Transforming qualitative information: thematic analysis and code development.

[52] Mayring P (2000). Qualitative content analysis. Forum Qualitative Sozialforschung/ Forum. Qual Soc Res.

[53] QSR International (2015). NVivo professional version 11.

[54] Babbie ER (2013). The practice of social research.

[55] Anderson M, Elam G, Gerver S, Solarin I, Fenton K, Easterbrook P (2010). “It took a piece of me”: initial responses to a positive HIV diagnosis by Caribbean people in the UK. AIDS Care.

[56] Greeff M, Phetlhu R, Makoae LN, Dlamini PS, Holzemer WL, Naidoo JR, Kohi TW, Uys LR, Chirwa ML (2008). Disclosure of HIV status: experiences and perceptions of persons living with HIV/AIDS and nurses involved in their care in Africa. Qual Health Res.

[57] Simbayi LC, Kalichman SC, Strebel A, Cloete A, Henda N, Mqeketo A (2007). Disclosure of HIV status to sex partners and sexual risk behaviours among HIV-positive men and women, Cape Town, South Africa. Sex Transm Infect.

[58] Hult JR, Maurer SA, Moskowitz JT (2009). “I'm sorry, you're positive”: a qualitative study of individual experiences of testing positive for HIV. AIDS Care.

[59] de-Graft Aikins A, Akyeampong E, Hill AG, Kleinman AM (2015). Mental illness and destitution in Ghana: A Social-psychological perspective. The Culture of Mental Illness and Psychiatric Practice in Africa.

[60] Baumgartner LM, David KN (2009). Accepting being poz: the incorporation of the HIV identity into the self. Qual Health Res.

[61] NAP+, GAC and UNAIDS (2014). Persons living with HIV Stigma Index Study, Ghana.

[62] Baiden F, Baiden R, Williams J, Akweongo P, Clerk C, Debpuur C, Philips J, Hodgson A (2005). Review of antenatal-linked voluntary counseling and HIV testing in sub-Saharan Africa: lessons and options for Ghana. Ghana Med J.

[63] Gadegbeku C, Saka R, Mensah B (2013). Attitude of the Youth towards Voluntary Counselling and Testing (VCT) of HIV/AIDS in Accra, Ghana. J Biol Agri Healthcare.

[64] Oppong AK (2013). HIV/AIDS knowledge and uptake of HIV counselling and testing among undergraduate private university students in Accra, Ghana. Reprod Health.

[65] Tenkorang EY, Owusu AY (2013). Examining HIV-related stigma and discrimination in Ghana: what are the major contributors?. Sex Health.

[66] Dapaah JM, Spronk R (2016). When the clinic becomes a home. Successful VCT and ART services in a stressful environment. J Soc Aspects HIV/AIDS.

[67] Badaru UM, Ogwumike OO, Adeniyi AF, Nelson EE (2017). Determinants of caregiving burden and quality of life of informal caregivers of African stroke survivors: literature review. Int J Disabil Human Dev.

[68] Omona J (2013). Sampling in qualitative research: improving the quality of research outcomes in higher education. Makerere J Higher Educ.

[69] Miles M, Huberman AM (1994). Qualitative data analysis: An expanded sourcebook.

[70] Peirce A (2017). The emotional impact of an HIV diagnosis.

[71] GAC (2016). National HIV & AIDS Strategic Plan 2016-2020.

[72] Owusu AY, Asante KT. Current HIV/AIDS status, access to antiretroviral treatment, and HIV related stigma in Ghana. Policy Brief. 2020. Accra: ISSER.

