# Supplementary material for: Experiences of new diagnoses among HIV-positive persons: implications for public health
Source: BMC Public Health. 2022 Mar 18;22:538. doi: 10.1186/s12889-022-12809-6 (PMC8932052; doi:10.1186/s12889-022-12809-6)
Supplement: Supplementary file 1 — Additional file 1. Qualitative/Indepth Interview Guide. Housing and Health Needs of Persons Living with HIV/AIDS Project. Open-ended qualitative indepth interview guide with sections A to G and ending with demographic/background data. [file 12889_2022_12809_MOESM1_ESM.docx]

Additional file 4

Supplementary File 1

**QUALITATIVE/INDEPTH INTERVIEW GUIDE**

**HOUSING AND HEALTH NEEDS OF PERSONS LIVING WITH HIV/ AIDS PROJECT**

**NOTE: *Respondents MUST HAVE BEEN OFFICIALLY/CLINICALLY DIAGNOSED AS HIV+ BEFORE INTERVIEING THEM***

**Section A: Consent to be interviewed:**

**Interviewer: Please seek the consent of the interviewee by reading the following to him/her and get his/her approval before beginning the interview:**

I am ---------- (INTERVIEWER: mention your name) from the University of Ghana. We are working together with Dr. Tenkorang, Dr. Adobea and Dr. Laar also from the University of Ghana on this study. The study is about the housing needs of HIV positive persons. We have realized that because of the health of such persons they should be given better housing conditions since they are easily infected by other diseases than others who don’t have HIV. We want to know some of the things that go on in your home so most of the questions I am about to ask you is about the situation in your house. Other questions are about your health and how you have been seeking healthcare for this disease/infection.

This study is important because it will help the Government know the kind of things persons with HIV/AIDS go through. It will also help the Government and other officials to know how best to help persons who are infected with HIV/AIDS.

It is not compulsory for you to talk to me regarding this study. If you do not talk to me, there will be no penalty (problem). However, I shall be grateful if you would talk to me. If you talk to me, we will not disclose your identity or any information you give me to anyone. That is, we will not let anyone know you were the one who spoke to me. All the information will be put together and used collectively for our reports for the good of all Ghanaians. So no one will know what you said specifically. Please will you like to speak to me?

**Please, note:** If the person does not agree to be part, thank him/her and leave. If she/he agrees to be part of the study, ensure her/him of confidentiality and have him/her sign the consent form (If she/he cannot read/write and wants to give a verbal consent in the presence of the person who accompanied him/her to the hospital, if any, they can do so. In the absence of a witness, they may also give a verbal consent to you.

INTERVIEWER: Please I will want you to sign this document for me. The document says your name and signature but if you are not comfortable you can give me just your signature to show that indeed you have accepted to speak with me. If you cannot sign, you can call anyone who accompanied you to the hospital to be a witness as you consent, or you may consent verbally to me.

The interview will take about one hour. Please can we start?

If you have concerns, email or call Dr. Owusu gaowusu@ug.edu.gh or (+233) (0)577699900

| Section C- PARTICIPANT AGREEMENT |
| --- |

**I have read or have had someone read all of the above, asked questions, received answers regarding participation in this study, and am willing to give consent for me, my child/ward to participate in this study. I will not have waived any of my rights by signing this consent form. Upon signing this consent form, I will receive a copy for my personal records.**

________________________________________________

Name of Participant

_________________________________________________

Signature or mark of Participant Date

**If you (participant) cannot read and or understand the form yourself, a witness must sign here:**

I was present while the benefits, risks and procedures were read to the volunteer. All questions were answered and the volunteer has agreed to take part in the research.

_________________________________________________

Name of witness

________________________________________________

Signature of witness/Mark Date

I certify that the nature and purpose, the potential benefits, and possible risks associated with participating in this research have been explained to the above individual.

__________________________________________________

Name of Person Who Obtained Consent

___________________________________________

Signature of Person Who Obtained Consent Date

Thank you for agreeing to be part of this study.

*Confirm that the respondent has been diagnosed officially with HIV!*

*Please, I will like to know when you were diagnosed with HIV (year and month): ......................*

**Section B: Housing arrangements/type**

Do you currently live in a house **(i.e., not homeless?)**

a. Type of housing (Own house, Rented house, Family House, Uncompleted building, homeless etc.). What aspects of this type of housing do you particularly like? Why? What aspects of this type of housing you dislike? Why?

b. Housing arrangement (Shared/not shared, self-contained but semi-detached, self-

contained/single household accommodation, detached house, ‘boys’-quarters’, non-residential building (church, school classrooms, kiosk, container, shop, incomplete house, etc.) (PROBE: what about it do you like? Not like?)

c. Length of stay in current dwelling (PROBE: how has your experience been like? What works? What isn’t working right now? What is the difference between this current dwelling/house and your last house? (PROBE: warmth, space, privacy, damp, ventilation, lighting etc.)

How long have you lived in this same house and why? What has been your experience living here?

Where did you live when you were first diagnosed with HIV by a physician? What has been your experience?

What would your housing preference be in the future? What are the reasons for this preference?

What are the reasons for wanting to remain in the same house?

In your opinion, how would you describe your experience with the following living arrangement in your current dwelling/house:

a. Alone as an HIV patient? How does it feel like living alone? Would you prefer living alone now and in the future? why

b. With family members (nuclear or extended). How well do they relate to you? What are your expectations of them? Would you prefer living with them? Why?

c. With other people/tenants who have been diagnosed with HIV? What do you like about that? What is it you don’t like about?

d. With other people/tenants who don’t have HIV? (How will you describe their behaviour towards you?

Who are the people you lived with before you were diagnosed with HIV? How will describe your experience with them?

What type of housing would be you prefer now or in the future? (PROBE: tenure type, type of dwelling/house, size and location)

What have been your experiences with housing since you were diagnosed with HIV? Please, tell me more (PROBE:

a. what kinds of problems have you encountered in getting housing?

b. Have you ever been evicted or asked to leave? Why? How did things turn out?

c. Have you ever experienced discrimination/stigma? How will you describe it (meaning)? How did it happen? How did you deal with it?

d. How does the current housing situation affect your adherence to treatment? What are the barriers to treatment?

What are the conditions in your current house (PROBE):

a. What amenities/facilities have been helpful to you? Which facilities? Why or why not?

b. How will you describe the conditions of the amenities/facilities?

c. Are there any facilities/amenities you will like which are not available? Which facilities? why?

What are your views about housing for HIV/AID patients in terms of:

a. It’s location (proximity to family, services, amenities)

b. The mix of residents (gender, ethnicity, health status)

c. The type of support available

d. Size (number of rooms)

e. Communal facilities (kitchen, toilet, bathroom etc.)

f. Provision of health and social care

Is there anything that worries you as an HIV patient about your current or future housing situation? (PROBE overcrowding, tenure insecurity, sanitation problems, lack of basic services)

**Section C: SES and Housing**

If applicable, how much rent is paid for this house annually? Please state amount in Ghana Cedis

Who pays for the rent? Why?

Who pays for the utilities? Why?

How would you rate the affordability of this house?

If applicable: do people who live in the same house/household with you know of your HIV status? If not, why not?

Do you find that you are discriminated against/stigmatized with regards to housing issues due to your HIV status?

Does HIV/AIDS serostatus in any way compromise your chances of living in adequate and affordable homes? If yes, how?

Do you think your HIV status affects your chances/ability to live in adequate and affordable housing in any way? Please tell me more.

PROBE: For instance, are you denied adequate and affordable housing because of your serostatus?

Are you denied access to some part of the house/home because of your HIV status? If yes, which part(s), who restricts your access, and why?

If yes, which area(s) of the home are you restricted from? Why?

Is your housing conditions affected because you are too sick to work? How?

PROBE, IF NECESSARY: Specifically, are you unable to afford adequate home/space in the home because you are too sick to work? Why do you think so?

Have you ever experienced eviction from your house?

If yes, how many times has the eviction happened?

If yes, what happened and why did it happen?

Do you think it is connected to you HIV status? Why?

**Section D: Section for PLHIV who are homeless (by the time of interview)**

(If the person is homeless, this is also an important information, and **do capture that**)?

Note: If the person is homeless, ask him/her why he/she is homeless, ......... and for how long...

Ask about the circumstances that led to his/her homelessness......

Ask about where he/she spends most of her time and why

Ask him/her what he/she thinks have to be done to enable him/her live in a home/house again.

**Section E: Social Support Needs/Assistance**

Do you currently have support needs and if so who caters for these? (PROBE: immediate family members, extended family members, other tenants, neighbours, agencies (government/NGO). What type of support? What did you like about these support services from the provider? Why? What is not working well with this support services? Why? In your opinion, what can be done to improve on these support?

**Section F: Housing and Health**

How would you describe your health generally?

Has your current housing arrangement had effects on you in any way? If yes, what reasons do you think are attributed to these changes?

Has the type of housing and its conditions prevented you from taking (better) care of your HIV status in any way? What are the challenges? (PROBE: to take medicines, cook good food, go to the hospital, practise safe sex, etc.)

Does your current housing situation (for instance where it is situated—distance, etc.) prevent you from going to the hospital/clinic/pharmacy to seek healthcare? How, why?

Where do you go for your HIV/AIDs care? (PROBE: hospital, clinic, health center). What services have you had that had been helpful to you? Why? Has it been difficult to get the services you need? What are the difficulties you have experienced?

In what ways do you feel your family members/tenants are helpful/supportive of your visits to the hospital/clinic/health center?

What are the problems with your physical health? Why? How does your physical health impact on your daily life?

What has been your experience with the following:

a. Anxiety

b. Depression

c. Isolation

**Section G: Reactions towards initial HIV/AIDS diagnosis**

What was your experience when you were diagnosed/how did you receive the news for the first time you were told you had HIV?

Probe:

- How did you feel immediately?
- How did you take in that message?
- How did you react?
- What came to mind immediately you were first told you had HIV?

Why did you react this way?

What influenced/prompted/informed your immediate reaction to the news, as you have described to me?

Probe:

- Did you expect some help/difficulties/ease with managing the situation?
- Do you think this influenced your first reaction to the news?
- Did anything/anyone influence you/your first reaction to the news?
- If yes, what and how?

Certainly, you have lived through it. What particularly/main factor(s)/setting(s)/issue(s) helped you manage/move on from your first reaction to how you feel/what you are doing/where you are now?

Probe:

- Did anybody/anything help?
- If yes, who/what?
- How, why?

Please, tell me more. How did such things/persons help you get to the point where you are presently coping with/managing the disease?

**Demographic/Background Data**

Respondent’s ID

Age of respondent:

Marital status of respondent

Number of his/her biological children who are alive and live with her

Number of other children for whom she/he may be a guardian (non-biological children he/she is responsible for) and live in the same dwelling with her/him

Level of Education completed:

Ethnicity: Akan, Ga/Adangbe, Ewe, Northern Ethnic groups

Religious affiliation: Christians, Muslims, Traditionalists, No religion

Gender: Male, Female

Sexual orientation: opposite-sex relationship(s), Lesbian, Gay, Bisexual

Residence: Rural, Urban

Employment status:

Type of employment: Self-employed, Private sector, Public sector, Other

If he/she is currently unemployed, what are the reasons for this?

Number of persons in his/her household and their ages (in completed years)
